# Human Herpesvirus 8 infection may contribute to oxidative stress in diabetes type 2 patients

**DOI:** 10.1186/s13104-020-4935-3

**Published:** 2020-02-13

**Authors:** Alessandra Incani, Luisa Marras, Gabriele Serreli, Angela Ingianni, Raffaello Pompei, Monica Deiana, Fabrizio Angius

**Affiliations:** 1 Unit of Experimental Pathology, Department of Biomedical Sciences, University of Cagliari, Cagliari, Italy; 2 Section of Applied Microbiology, Department of Biomedical Sciences, University of Cagliari, Cagliari, Italy

**Keywords:** Oxidative stress, Diabetes type 2, Human Herpesvirus 8

## Abstract

**Objective:**

To investigate the link between Human Herpesvirus 8 (HHV8) infection and plasma oxidative stress in patients with diabetes mellitus type 2 (DM2).

**Results:**

Blood samples collected from DM2 and control subjects were screened for the presence of antibodies against HHV8 and for biomarkers of oxidative stress. We determined the products of radical damage on the plasma lipid fraction, such as malondialdehyde (MDA), fatty acid hydroperoxides (HP) and 7-ketocholesterol (7-keto), the oxidation products of unsaturated fatty acids (UFA) and cholesterol, respectively. The level of plasma antioxidant α-tocopherol (α-toc) was also assessed. Relevant differences were observed in the redox status in DM2 and either HHV8-positive or -negative control subjects. The level of α-toc significantly decreased in both DM2 and HHV8-positive subjects. Levels of MDA, HP and 7-keto were much higher in HHV8-positive and DM2 subjects, indicating that plasma oxidative stress is a common feature in both DM2 and HHV8-infection. In addition, 7-keto was further increased in HHV8-positive DM2 patients. We hypothesized that the HHV8-infection may contribute to the production of ROS, and hence to the oxidative stress closely related to the pathogenesis and development of DM2.

## Introduction

The Human Herpesvirus 8 (HHV8), known as the causative agent of Kaposi’s sarcoma (KS), establishes a persistent latent-infection for the host’s lifespan with occasional reactivation of the acute infection [1]. The HHV8 latency-associated nuclear antigen (LANA) is known to be able to immortalize primary endothelial cells and enhance cell survival in critical conditions [2]. Several studies have demonstrated that HHV8 infection induces intense and long-lasting alterations in the physiology of infected cells [3–5]. HHV8 has also been associated to widely diffused chronic diseases [6–12], such as cardiovascular disease and diabetes mellitus type 2 (DM2). HHV8 induces a permanent inflammatory condition with impairment of B-lymphocyte activity and alteration in the function of NK-cells [13, 14], as also found in DM2 patients. HHV8 has recently been reported as inducing reactive oxygen species (ROS) production both during the very early phase of infection—efficiently facilitating viral entry into the micro-vascular cells via macro-pinocytosis—and during the establishment of latency in endothelial cells leading to junction dysregulation and increased vascular permeability [13, 15]. Moreover, ROS induced by HHV8 play a causal role in KS malignancies by promoting proliferation and angiogenesis that have been shown to be efficiently reduced by the antioxidant N-acetylcysteine in animal models [16]. It is noteworthy that ROS play a pivotal role in the metabolic modifications induced by DM2 [16–20] and that the agents which increase redox activity or generate ROS result in the stimulation of the basal insulin secretion [13, 21, 22], hence revealing their involvement in the initiation and progression of DM2 [23]. Increased free radicals production in DM2 has also been shown to alter and induce several risk factors for cardiovascular diseases such as lipid peroxidation, endothelial dysfunction, inflammation and platelet activation [24]. Lipid peroxidation, which affects low-density lipoprotein (LDL), is considered to play an important role in the atherosclerosis of DM2 patients [25]. Oxidation of lipoproteins induces various changes in their lipid composition, with a substantial loss of free and esterified cholesterol, fatty acids and co-occurrence of their oxidation products [26]. Products of lipid peroxidation like malondialdehyde (MDA) [27, 28] and oxysterols such as 7-ketocholesterol (7-keto) have been detected in DM2 patients [29, 30]. In this study we focused on the role of HHV8-infection in the alteration of the plasma redox status of a DM2 cohort and compared to that of control subjects. As biomarkers of oxidative stress, we determined the more stable products of radical damage on the plasma lipid fraction, such as MDA, and more sensible and precise markers of the lipid peroxidation process, such as fatty acid hydroperoxides (HP) and 7-keto, oxidation products of unsaturated fatty acids (UFA) and cholesterol, respectively. The level of plasma lipophilic antioxidant α-tocopherol (α-toc) was also detected, since its level is generally correlated to that of peroxides and aldehydes in the plasma of DM2 patients [31].

## Main text

### Materials and methods

#### Serological analysis

DM2 patients and a control group of non-DM2 volunteers (ascertained by the OGTT test), matched for age (44–70 years) and sex, were recruited at the Diabetes and Metabolic Diseases Service (San Giovanni City Hospital, Cagliari). DM2 diagnosis was performed according to World Health Organization (WHO) criteria for the classification of diabetes [32], based on a fasting glucose level above 7 mmol/L verified on at least two occasions (i.e. on the basis of the clinical documentation and the OGTT test). Subjects with tumours or infectious diseases, aged over-70 or pregnant were excluded from the study. Experimental protocols involving human subjects and sample collection were performed following the guidelines approved by the Local Ethical Committee and were subordinate to the acquisition of informed consent from all participants which was then anonymized before use [10, 32, 33]. About 10 mL samples of venous blood were drawn after 12 h fasting, centrifuged to separate plasma and then stored in different tubes at − 80 °C until serological analysis. The samples from DM2 patients (N. 31; 15 HHV8-positive and 16 HHV8-negative) and control subjects (N. 23; 9 HHV8-positive and 14 HHV8-negative) were screened for the presence of anti-HHV8 antibodies using a validated immunofluorescence kit assay (Scimedx Corp., Denville, NJ, USA), following the manufacturer’s instructions. Only plasma with an antibody titre higher or equal to 1:64 was considered positive. In addition, the presence of latent HHV8-DNA was detected in both diabetics and controls by a PCR method as described elsewhere [3, 33].

#### Characterization of the oxidative status

In order to evaluate the plasma oxidative status, we measured the level of α-toc, MDA, 7-keto and HP, cholesterol and UFA oxidation products, in HHV8-positive and -negative samples from controls and DM2 subjects. Total lipids were extracted from the plasma samples (150 μL) through a CHCl_3_/MeOH (2/1 v/v) solution and separated by mild saponification [34]. Cholesterol, UFA and their oxidation products were identified and quantified by an HPLC system (Agilent Technologies, Santa Clara, CA) equipped with a diode-array detector (HPLC–DAD). Cholesterol, detected at 203 nm, and 7-keto, detected at 245 nm, were measured using a Varian column (Middelburg, The Netherlands), Inertsil 5 ODS-3, 150 × 3 mm, with MeOH as the mobile phase, at a flow rate of 0.4 mL/min. UFA, detected at 200, and HP, detected at 234 nm, were measured using a Varian column, Inertsil 5 ODS-2, 150 × 4.6 mm, with a mobile phase of CH_3_CN/H_2_O (70/30, v/v) containing 0.12% CH_3_COOH, at a flow rate of 1.5 mL/min. α-tocopherol was determined by HPLC-electrochemical detection (DECADE II, Antec) set at an oxidizing potential of 0.6 V, using a C-18 Hewlett Packard ODS Hypersil column, 5 μm particle size, 100 × 2.1 mm, with a mobile phase of MeOH/CH_3_COONa 0.05 M pH 5.5 (95/5 v/v) at a flow rate of 0.3 mL/min [34]. The MDA level was directly measured in the plasma samples by the TBARS test with HPLC–DAD quantification. Briefly, 100 μL of TCA 10% were added to 30 μL of plasma diluted in 370 μL of a water/MeOH solution (40/60 v/v), then samples were mixed and left at room temperature. After 20 min, 200 μL of TBA (0.6%) were added; samples were incubated at 90 °C for 45 min and then centrifuged at 5000×*g* for 15 min at 4 °C. Aliquots of the supernatant were used for HPLC–DAD analysis, using a Varian column, Inertsil 5 ODS-2, 150 × 4.6 mm, and a mixture of KH_2_PO_4_ 50 mM pH 7/MeOH (65/35, v/v) was used as mobile phase at a flow rate of 1 mL/min. The adduct MDA-TBA was revealed at 532 nm [35, 36].

### Statistics

Statistical analysis was performed with GraphPad Prism 7 software (La Jolla, CA, USA). All data were expressed as the mean ± SEM of experiments in triplicate and analysed by the t-student test or one-way Analysis of Variance (ANOVA) and Bonferroni as post hoc test for multiple comparisons when required. Differences were considered significant when p < 0.05.

### Results

**Fig. 1:**
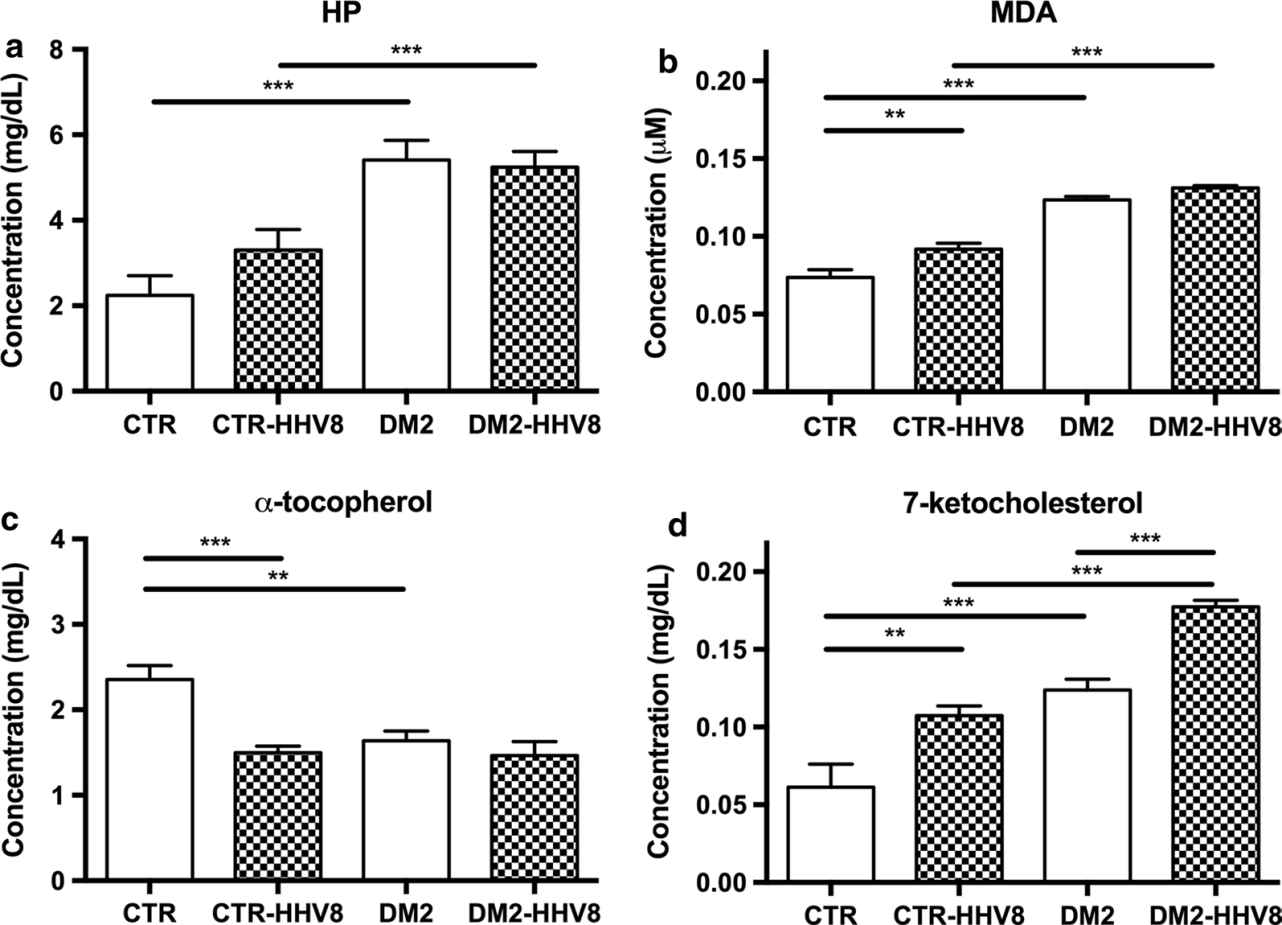
Plasmatic concentrations of fatty acid hydroperoxides, malondialdehyde, α-tocopherol and 7-ketocholesterol in control and DM2 subjects. **a** Fatty acid hydroperoxides (HP), **b** Malondialdehyde (MDA), **c** α-tocopherol and **d** 7-ketocholesterol were extracted from plasma samples, separated, identified and quantified by HPLC as reported in “Materials and methods” section. HP shows a significantly higher concentration in DM2 subjects as compared to non-diabetic controls (p < 0.001). MDA was much higher in DM2 patients versus controls (p < 0.001) and significantly even higher in DM2 subjects positive for HHV8 compared to HHV8-negative DM2 (p < 0.01). Whereas α-tocopherol shows a decrease in both DM2 and HHV8 (either positive or negative) subjects (p < 0.01), 7-ketocholesterol was significantly higher in all the HHV8-positive samples (p < 0.01); there was a general increase in 7-ketocholesterol in DM2 subjects versus non-DM2 controls. A further significant increase in 7-ketocholesterol (p < 0.001) was detected in DM2 HHV8-positive samples. The data are expressed as the mean concentration values + SEM and significance is indicated with (*) when p < 0.05, (**) when p < 0.01 or (***) when p < 0.001, as calculated by ANOVA and Bonferroni as post hoc tests. *CTR* non-diabetic control subjects, *DM2* diabetic subjects, *HHV8* infected subjects (patterned bars)

**Fig. 2:**
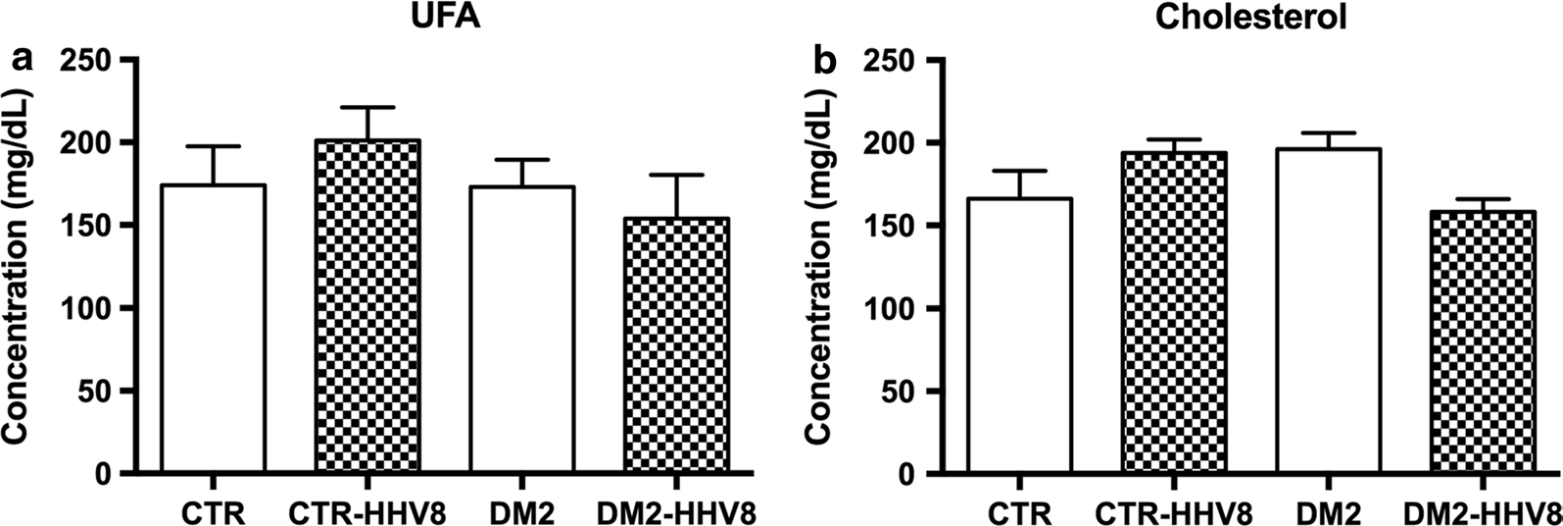
Lipid concentrations in control and DM2 subjects. **a** Unsaturated fatty acids (UFA) and **b** cholesterol were extracted from plasma samples, separated, identified and quantified by HPLC as reported in “Materials and methods” section. No differences in UFA or cholesterol were found between DM2 and controls, either HHV8-positive or -negative. The data are expressed as the mean concentration values + SEM and significance was calculated by ANOVA and Bonferroni post hoc tests. *CTR* non-diabetic control subjects, *DM2* diabetic subjects, *HHV8* infected subjects (patterned bars)

A significant increase in HP was found in DM2 subjects as compared to non-diabetic controls (Fig. 1a, p < 0.001), whilst no significant differences were observed between HHV8-positive and -negative DM2 patients (Fig. 1a). MDA was remarkably higher in DM2 versus either HHV8-positive and HHV8-negative controls (Fig. 1b, p < 0.001); in addition, MDA significantly increased in HHV8-positive controls versus HHV8-negative ones (p < 0.01). No difference was found between DM2 and DM2 HHV8-positive subjects. The α-toc showed an overall decrease in all DM2 samples (p < 0.01) and HHV8-positive non-DM2 samples also revealed a remarkable decrease compared to HHV8-negative control samples (Fig. 1c, p < 0.01). 7-keto appeared significantly enhanced in all the HHV8-infected subjects (Fig. 1d, p < 0.01) irrespective of the presence of DM2. However, there was also a general increase in 7-keto in DM2 subjects as compared to non-diabetic controls (p < 0.01). Strikingly, in HHV8-positive samples from DM2 subjects we found a significant further increase in 7-keto (p < 0.001) compared to HHV8-negative DM2 samples. No significant differences were observed for cholesterol and UFA in all the experimental groups (Fig. 2).

### Discussion

It is widely accepted that ROS play a pivotal role in DM2 both in the early stages, when insulin resistance is being set up and, later on, when complications occur. ROS cause insulin resistance in the peripheral tissues by affecting various points in insulin receptor signal transduction [37]. As a matter of fact, the production of an unusual amount of ROS can generate systemic oxidative stress, which can directly damage tissues or stimulate the production of inflammatory cytokines with subsequent cell damage and even apoptosis in pancreatic β-cells [38, 39]. Furthermore, some authors have underlined the possibility that any factor able to induce either acute or chronic hyperglycaemia may trigger ROS production, which causes systemic inflammation, ER stress and diabetic complications [21, 40, 41]. Unfortunately, the strategies to directly control hyperglycaemia, through diet and/or specific pharmacological therapies, are not always sufficient to avoid the occurrence of complications in diabetic patients, thus the control of risk factors is still the best approach to reduce the incidence and complications of DM2. In this scenario, the large amount of evidence of high rate of classic KS, HHV8 genome and sero-positivity in DM2 patients described in the last decades [7, 10, 42, 43], and also the recent findings about the possible role/cooperation of ROS, induced by HHV8, in endothelial dysregulation [13, 15], all support the idea that, in order to persist in the host, HHV8 implements strategies which can lead to chronic pathological implications [11].

In this work we observed and corroborated significant differences in the plasma oxidative status between control and DM2 subjects, who were either positive or negative for HHV8: the MDA level significantly increased in samples from diabetic subjects, as shown in previous studies [27, 28], and a significant concentration of HP and 7-keto was also detected, highlighting an extended lipid peroxidation process, triggered by ROS. Although there are few studies related to oxysterols in DM2 [30], all of them underline a significant increase in these products, which are considered important biomarkers of oxidative stress and mainly originate in the systemic circulation during LDL oxidation [44, 45]. As expected, the level of the antioxidant α-toc was lower in DM2 subjects compared to controls, further indicating a condition of oxidative stress. Simultaneous detection of lipid oxidation products and α-toc is relevant for studying the oxidative stress/antioxidant balance at the plasma level.

Interestingly, these biomarkers showed the same trend when measured in the plasma of HHV8-positive individuals, indicating a similar condition of oxidative stress. The level of HP, 7-keto and MDA were significantly higher in DM2 with respect to the controls. Strikingly, both the MDA and 7-keto levels showed a further increase in HHV8-positive DM2 subjects compared to the HHV8-negative ones, supporting the idea that the HHV8-infection itself may contribute to oxidative stress—confirmed by the lower α-toc found in infected controls as compared to uninfected ones—and hence to tissue damage [13, 15–17]. In fact, plasma lipid oxidation products contribute to the endothelial cell dysfunction that characterizes the onset of atherosclerotic plaque [46]. In particular, oxysterol 7-keto has been shown to exhibit both pro-inflammatory and cytotoxic properties that lead to atherosclerosis. 7-keto induces a clear inflammatory phenotype in human endothelial cells [47] and foam cell formations [48]; it enhances the expression of the vascular endothelial growth factor (VEGF) [49], decreases NO-induced vascular relaxation [50, 51] and induces apoptosis in smooth muscle cells [52]. It therefore sounds reasonable to speculate that the presence of 7-keto in HHV8-positive patients, as in those with DM2, may be indicative of a pro-atherogenic and pro-inflammatory environment, which will likely lead to the development of atherosclerosis and cardiovascular complications. Our results corroborate the assumption that DM2 is associated to plasma oxidative stress [20] and support a similar condition in HHV8-positive subjects wherein the HHV8-infection, by inducing abnormal ROS production, most probably contributes to causing and/or maintaining a condition of oxidative stress.

## Limitations

The findings reported here should be considered within the context of the study’s limitations.

- Although reliable methods were used, the work does have several levels of limitation, the greatest of which is the low number of patients and hence the sample size tested. This fact was also due to the difficulty in finding HHV8-positive controls.
- Moreover, this limit is further impacted by the fact that the samples came from a single city hospital, which results in a variability weakness.
- These are preliminary data focused on plasma oxidative status. In a larger sample size, other plasma antioxidant defences than α-toc should also be evaluated, which could help in providing a complete picture of plasma redox status.

## Data Availability

All data generated or analysed during this study are included in this published article.

## Abbreviations

HHV8: Human Herpesvirus 8
KS: Kaposi’s sarcoma
LANA: Latency-associated nuclear antigen
ROS: Reactive oxygen species
DM2: Diabetes mellitus type 2
α-Toc: α-Tocopherol
UFA: Unsaturated fatty acids
HP: Fatty acid hydroperoxides
7-Keto: 7-Ketocholesterol
MDA: Malondialdehyde
